# Imaging receptor for advanced glycation end product expression in mouse model of hind limb ischemia

**DOI:** 10.1186/2191-219X-3-37

**Published:** 2013-05-11

**Authors:** Yared Tekabe, Maria Kollaros, Chong Li, Geping Zhang, Ann Marie Schmidt, Lynne Johnson

**Affiliations:** 1 Department of Medicine, Columbia University Medical Center, 622 West 168th St, PH 10 room 203, New York, NY 10032, USA; 2 Department of Pathology, Columbia University Medical Center, New York, NY 10032, USA; 3 Department of Medicine, New York University Medical Center, New York, NY 10016, USA

**Keywords:** Limb ischemia, Diabetes, RAGE, Molecular imaging, Radionuclides

## Abstract

**Background:**

The purpose of this study is to image the effect of diabetes on expression of receptor for advanced glycation endproducts (RAGE) in limb ischemia in live animals.

**Methods:**

Male wild-type C57BL/6 mice were either made diabetic or left as control. Two months later, diabetic and non-diabetic mice underwent left femoral artery ligation. The right leg served as lesion control. Five days later, mice were injected with 15.1 ± 4.4 MBq ^99m^Tc-anti-RAGE F(ab’)_2_ and 4 to 5 h later (blood pool clearance) underwent SPECT/CT imaging. At the completion of imaging, mice were euthanized, hind limbs counted and sectioned, and scans reconstructed. Regions of interest were drawn on serial transverse sections comprising the hind limbs and activity in millicuries summed and divided by the injected dose (ID). Quantitative histology was performed for RAGE staining and angiogenesis.

**Results:**

Uptake of ^99m^Tc-anti-RAGE F(ab')_2_ as %ID × 10^−3^ was higher in the left (ischemic) limbs for the diabetic mice (*n* = 8) compared to non-diabetic mice (*n* = 8) (1.20 ± 0.44% vs. 0.49 ± 0.40%; *P* = 0.0007) and corresponded to less angiogenesis in the diabetic mice. Uptake was also higher in the right limbs of diabetic compared to non-diabetic animals (0.82 ± 0.33% vs. 0.40 ± 0.14%; *P* = 0.0004).

**Conclusions:**

These data show the feasibility of imaging and quantifying the effect of diabetes on RAGE expression in limb ischemia.

## Background

Peripheral artery disease (PAD) is a common condition producing symptomatic ischemic leg pain with exertion (claudication) and in diabetics can be a particularly malignant disease leading to rest pain and gangrene requiring amputation. There are currently no effective drug therapies for symptomatic PAD, leaving surgical revascularization and interventional catheter-based approaches. There is a wealth of published data from experimental studies and a few clinical studies that provide evidence to support the central role for receptor for advanced glycation endproducts (RAGE) in the development and progression of PAD in atherosclerosis and diabetes.

RAGE is a 35 kDa polypeptide of the immunoglobulin superfamily that is a multiligand receptor shown to be an important mediator of inflammation in a variety of conditions including atherosclerosis and the complications of diabetes [1-7]. RAGE is constitutively expressed at low levels on smooth muscle cells and endothelial cells in the vascular endothelium [3]. Increased expression of RAGE in the vascular wall occurs in response to a number of stimuli including hyperlipidemia and hyperglycemia [3-6]. In the non-diabetic limb, in response to tissue hypoxia, via the hypoxia-inducible factor-1 alpha (HIF-1a) pathway, vascular endothelial growth factor (VEGF) is released locally and stimulates angiogenesis through multiple mechanisms that include increased proliferation and decreased apoptosis of endothelial cells and chemoattraction of monocytes into the ischemic tissue [8,9]. In diabetes, this normally adaptive response to hypoxia is blunted. RAGE has been shown to have a major mechanistic role in this maladaptive response by reducing VEGF mRNA and by inducing a defect in signal transduction resulting in fewer monocytes in the tissue to stimulate angiogenesis [10-12]. Experimental studies suppressing RAGE ligands have shown improvement in angiogenic response to limb ischemia [13,14].

We have previously shown that molecular imaging using a radionuclide probe targeting ανβ3 expression during angiogenesis can detect the effect of diabetes to suppress angiogenesis in mouse models of hind limb ischemia [15]. We have developed a radiolabeled murine monoclonal antibody targeting RAGE for *in vivo* imaging of RAGE expression and have shown focal uptake of this probe in atheroma in apolipoprotein E-deficient (apoE^−/−^) mice both with and without diabetes [16]. In this current study, we extend our previous work to investigate the hypothesis that RAGE-directed molecular imaging can detect the effect of diabetes on RAGE expression in a murine model of hind limb ischemia.

## Methods

All animal experiments were performed in accordance with the approval of the Institutional Animal Care and Use Committee of Columbia University. Male wild-type (WT) C57BL/6 mice (*n* = 20) were obtained (Jackson Laboratories, Bar Harbor, ME, USA). Diabetic mice (*n* = 8) and non-diabetic mice (*n* = 8) were injected with radiolabeled anti-RAGE F(ab’)_2_. Probe control non-diabetic mice (*n* = 4) were injected with non-immune IgG F(ab’)_2_.

### Induction of diabetes

At 6 weeks of age, mice were treated with streptozotocin (STZ; Sigma-Aldrich Corporation, St. Louis, MO, USA). Animals were injected with five consecutive daily doses of STZ dissolved in citrate buffer (55 mg/kg, pH 4.5) via the intraperitoneal route. One week after the first dose, blood glucose levels were assessed using a glucometer. The criterion of two consecutive glucose levels >250 mg/dL was used to indicate diabetes. If glucose levels were <250 mg/dL, then the mice received two additional doses of STZ. Both diabetic and non-diabetic mice were followed for 2 months to allow the diabetes to stabilize before femoral artery ligation.

### Femoral artery ligation

The hair on the abdominal wall and pelvis and the upper legs was shaved using an electronic shaver after the mouse was under anesthesia with 4% isoflurane induction and 1% maintenance. The skin was prepped using povidone-iodine (5%) followed by three alcohol preps. A skin incision was made on the upper thigh of the mouse. The inguinal ligament and the upper half of the femoral artery are exposed in both legs. On the left leg, the femoral artery was ligated with two sterile 8/0 non-absorbable silk sutures below the inguinal ligament proximally and just above the bifurcation into the superficial and deep femoral arteries distally. The vascular bundle on the right leg was isolated without further intervention. The skin incision was closed with sterile 5/0 nylon suture.

### Preparation of radiotracer

Monoclonal anti-RAGE antibody was developed as previously described [16]. For ^99m^Tc labeling, the antibody was fragmented into F(ab’)_2_ using a pepsin digestion kit (Pierce, Rockford, IL, USA). Approximately 1 mg of the F(ab’)_2_ was conjugated with 5 M excess of diethylene triamine pentaacetic acid (DTPA; Sigma). The reaction mixture was incubated at room temperature for 30 min followed by overnight dialysis at 4°C in phosphate-buffered saline (PBS) (0.15 M NaCl, 0.05 M NaHCO_3,_ pH 7.6). To 50 to 100 μg of anti-RAGE F(ab’)_2,_ 50 μg of SnCl_2_ in 0.1 N HCl (flushed in N_2_ gas for 15 min) and 30 to 50 mCi ^99m^Tc were added and incubated at room temperature for 45 min. The ^99m^Tc-labeled antibody fragments were separated from free ^99m^Tc using a PD-10 column pre-equilibrated with 0.1 M PBS (pH 7.4). The mean radiopurity was 97 ± 0.5% by instant thin-layer chromatography. Control non-specific mouse IgG F(ab’)_2_ was similarly conjugated with DTPA for ^99m^Tc labeling as described above.

### *In vivo* imaging

Five days after femoral artery ligation, each mouse was anesthetized with isofluorane (4% to induce, 1% to maintain) for placement of a jugular vein catheter (Braintree Scientific, Braintree, MA, USA) and was injected with 0.41 ± 0.12 mCi ^99m^Tc-anti-RAGE F(ab’)_2_ or control non-specific mouse IgG F(ab’)_2_ and 4 to 5 h later (blood pool clearance) underwent single-photon emission computed tomography/computed tomography (SPECT/CT) imaging on nanoSPECT/CT (Bioscan, Washington, DC, USA).

A topogram (sequence of 2D side view X-ray projections) was used to determine the axial scan range for SPECT and CT imaging. CT images were acquired with an integrated CT scanner using an X-ray tube at 45 kVp and an exposure time of 1,000 ms per view. Following CT acquisition, helical SPECT scans were acquired using dual-headed detectors, each outfitted with nine pinhole apertures. Each pinhole has a diameter of 1.4 mm with each collimator providing a transaxial field-of-view (FOV) of 30 mm and an axial FOV of 16 mm, extendable through helical scanning to 270 mm. SPECT data were acquired with the following parameters: step and shoot rotation, 30° step in 360° rotation using 24 projections, 60 s per projection, 256 × 256 frame size with 1.0-mm pixels, and 140 keV with 10% energy window. The obtained projection data were reconstructed by ordered subsets expectation maximization algorithm with the subset and iteration number set to 16 and 8, respectively, and a voxel size of 300 μm and SPECT and CT datasets fused.

At the completion of *in vivo* imaging, mice were euthanized by an intraperitoneal injection of pentobarbital (100 mg/kg).

### Image analyses

The scans were reconstructed and processed using InVivoScope software (Invicro, Boston, MA, USA). On serial 5-voxel-thick transverse sections from below hip joints to distal hind limbs, regions of interest were drawn and activity in mCi summed for each limb and subsequently divided by the injected dose (ID).

### Gamma well counting

The anterior tibialis muscles were dissected and weighed, and the radioactivity was determined in a gamma well counter (Wallac Wizard 1470, PerkinElmer, Waltham, MA, USA) and expressed as the percentage of injected dose per gram (%ID/g) of tissue. The radiotracer activity in the samples was corrected for background, decay time, and tissue weight.

### Histopathology

Three sets of explanted tibialis anterior muscles per group were fixed in 10% formalin for 48 h. Three serial sections (5 μm thick) per experiment from paraffin-embedded blocks were processed for hematoxylin and eosin (H&E) for morphological evaluation and immunohistochemical analysis.

Serial sections were deparaffinized and rehydrated followed by quenching of endogenous peroxidase activity with 0.3% hydrogen peroxide. Slides were then incubated overnight with monoclonal anti-RAGE antibody (50 μg/ml). Slides were incubated for 30 min with biotinylated secondary antibody. To identify capillary sprouting, staining was performed with biotinylated *Griffonia* (*Bandeiraea*) *simplicifolia* isolectin I (1:50; Vector Laboratories, Burlingame, CA, USA). Sections were treated for 30 min with VECTASTAIN ABC reagent (Vector Laboratories). Color reaction was visualized with 3′,3′-diaminobenzidine (DAB substrate kit, Dako, Mississauga, Ontario, Canada) and counterstained with Gill’s hematoxylin solution. All brown staining areas were counted for each of the three sections for both the left and right anterior tibialis muscles for each experiment, and averaged RAGE and lectin staining were quantified as area staining positive for the brown chromogen per 100× field.

Images were captured using a digital camera mounted on a Nikon microscope (Nikon Co., Tokyo, Japan) and analyzed using Image-Pro Plus software (Media Cybernetics Inc., Bethesda, MD, USA).

### Immunofluorescence

Dual fluorescent confocal microscopy studies were performed to identify the cell source of RAGE staining. Muscle sections (5 μm thick) were deparaffinized in xylene and incubated with monoclonal mouse anti-RAGE F(ab')_2_ (50 μg/ml; Texas Red) and co-stained with endothelial cells (FVIII, 1:200; fluorescein isothiocyanate), macrophages (Mac-3, 1:50; fluorescein isothiocyanate), and myocytes (anti-sarcomeric actin, 1:50; fluorescein isothiocyanate). The images were examined using a confocal fluorescence microscope (Nikon) and SPOT imaging software (Diagnostic Instruments, Inc., Sterling Heights, MI, USA).

### Statistical analysis

Continuous variables were expressed as mean ± standard deviation. Normality was assessed using the Sharpiro-Wilk *W* test. Equality of variances was assessed using Levene’s test. Comparisons between groups were made using paired two-tailed Student’s *t* test or the Mann–Whitney *U* test, as appropriate with *P* < 0.05 denoting significance. Correlation was assessed using the Pearson product–moment correlation coefficient. Statistical analyses were performed using STATA 10.1 (StataCorp, College Station, TX, USA).

## Results

### Scan analysis

**Figure 1:**
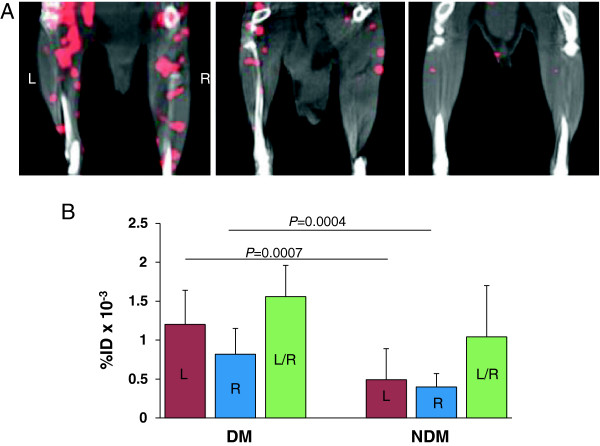
**Scan results.** (**A**) Representative coronal slices from SPECT/CT scans following injection of ^99m^Tc-anti-RAGE F(ab’)_2_ 5 days after left FAL for a WT diabetic mouse (left), WT non-diabetic mouse (center), and WT non-diabetic mouse injected with non-specific mouse IgG F(ab’)_2_ (right). (**B**) Bar graph shows quantitative RAGE uptake from scans in hind limbs for WT diabetic (left set of bars) and WT non-diabetic (right side set of bars) 5 days after FAL. Each bar represents average ± SD. DM, diabetes mellitus; NDM, non-diabetes mellitus; FAL, femoral artery ligation; WT, wild type.

Summed uptake of the probe on coronal slices from SPECT/CT scans following injection of ^99m^Tc-anti-RAGE F(ab’)_2_ 5 days after left femoral artery ligation showed greater uptake of the tracer in the left (ischemic) limbs of diabetic mice compared to the probe uptake in the ischemic limbs of non-diabetic mice injected with ^99m^Tc-anti-RAGE F(ab’)_2_ or in the ischemic limb of mice injected with non-specific IgG F(ab’)_2_ (control probe) (Figure 1A). The quantitative tracer uptake as %ID in the diabetic left (ischemic) limb (1.2 ± 0.44 × 10^−3^) was significantly higher than the uptake in the non-diabetic left limb (0.49 ± 0.40 × 10^−3^; *P* = 0.0007) (Figure 1B). Tracer uptake in the diabetic right (non-ischemic) limb (0.82 ± 0.33 × 10^−3^) was also significantly higher than the uptake in the non-diabetic right limb (0.40 ± 0.14 × 10^−3^; *P* = 0.0004).

### *Ex vivo* gamma counting

**Figure 2:**
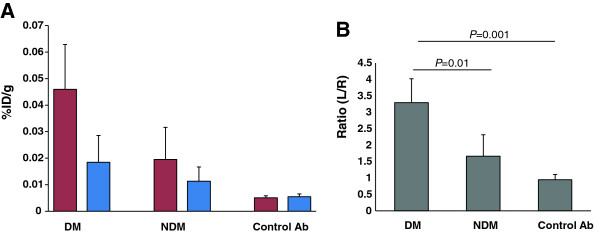
***Ex vivo *well counting for both hind limbs.** (**A**) Bars represent values for %ID/g. The red bars represent left (ischemic) hind limbs and the blue bars represent the right hind limbs. (**B**) Bars represent values for the ratios of left/right (L/R) limbs.

The higher uptake of ^99m^Tc-anti-RAGE F(ab’)_2_ in the diabetic left (ischemic) limbs was confirmed by *ex vivo* gamma well counting (Figure 2). The mean tracer uptake in the diabetic left limbs as %ID/g (0.046 ± 0.017) was significantly higher than the uptake in the non-diabetic left limbs (0.019 ± 0.012; *P* = 0.02) or limbs of mice injected with non-specific control IgG F(ab’)_2_ (0.005 ± 0.0007; *P* = 0004) (Figure 2A). The mean count ratio for left to right (L/R ratio) hind limbs for diabetic mice (3.29 ± 0.72) was also significantly higher than that for non-diabetic (1.66 ± 0.65; *P* = 0.01) or mice injected with control non-specific IgG F(ab’)_2_ (0.95 ± 0.15; *P* = 0.001) (Figure 2B).

### Histopathology

**Figure 3:**
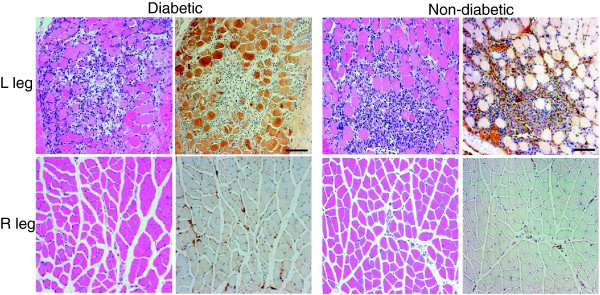
**Histological findings.** Serial sections from ischemic (L) diabetic and non-diabetic limbs. H&E staining is shown on the left and RAGE staining (brown) on the right for each set of images. Scale bar = 100 μm.

**Figure 4:**
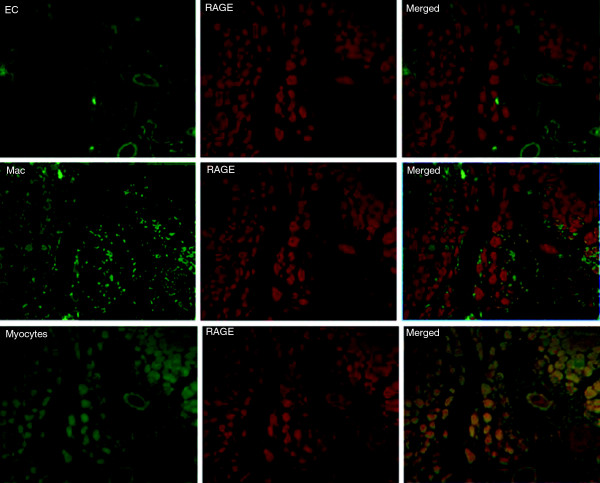
**Dual immunofluorescent staining for cells expressing RAGE in ischemic limb sections.** Sites of RAGE expression were shown to be mainly myocytes based on co-localization of RAGE (Texas Red) with anti-sarcomeric actin (green, fluorescein isothiocyanate) in the merged image. Co-localization of RAGE with macrophages (Mac-3, fluorescein isothiocyanate) was also seen in the merged image. Areas in yellow represent co-localization. EC, endothelial cells; Mac, macrophages.

Immunohistopathology supported the scan findings. Examples of anterior tibialis muscle tissue sections stained for H&E and RAGE are shown in Figure 3. The average RAGE staining, determined as percent area staining positive for the brown chromogen per 100× field, for the diabetic left limbs (21.3 ± 6.2%) was significantly greater than that for the contralateral right limb (5.6 ± 4.3%; *P* = 0.02) or non-diabetic left limbs (7.6 ± 5.6%; *P* = 0.04). Dual fluorescent staining identified RAGE co-localization predominantly with myocytes within the ischemic muscle (Figure 4).

**Figure 5:**
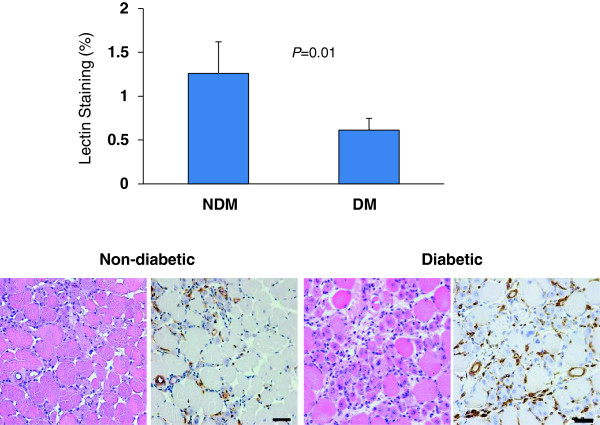
**Lectin staining.** Representative tissue sections obtained from ischemic hind limbs stained with H&E and lectin. Scale bar = 40 μm.

Serial sections were also stained for capillary sprouting (biotinylated *Griffonia* (*Bandeiraea*) *simplicifolia* isolectin I). Lectin staining for the non-diabetic left limbs (1.25 ± 0.36%) was significantly higher than that for the diabetic left limbs (0.61 ± 0.13%; *P* = 0.01) (Figure 5).

## Discussion

We demonstrated that the effect of diabetes on RAGE expression can be imaged and quantified in a murine femoral artery ligation model of limb ischemia using a RAGE-targeting ^99m^Tc-labeled probe and SPECT/CT imaging. The quantitative expression of RAGE from the scans correlated with the degree of suppression of angiogenesis measured from lectin staining on immunohistological sections of the ischemic and non-ischemic limbs. We have previously shown that the effect of diabetes to suppress angiogenesis in a similar live animal model can be imaged with ^99m^Tc-HYNIC-RGDs targeting ανβ_3_ integrin in ischemic limbs of diabetic mice with femoral artery ligation compared to non-diabetic mice [15]. The results of this current study show the potential feasibility of imaging and quantifying in living subjects another important biological signal in the development of peripheral arterial disease.

RAGE is a 35 kDa polypeptide of the immunoglobulin superfamily that is a multiligand receptor shown to play an important role in vascular disease [2-11]. This receptor is constitutively expressed in low levels on smooth muscle cells and endothelial cells in vascular walls [4]. RAGE binds ligands that induce expression and activation of signaling pathways important in promoting inflammation and reducing vascular reactivity. These ligands include advanced glycation endproducts (AGEs), S100/Calgranulin proteins, high-mobility group box 1 protein (HMGB1), and oxidized LDL. Ligand-RAGE binding activates intracellular pathways that generate reactive oxygen species, reduce nitric oxide availability, promote monocyte and leukocyte chemotaxis, and increase cytokine secretion [4-7]. Our finding of higher quantitative uptake of our radiolabeled probe in the contralateral (non-ischemic) limbs of the diabetic compared to the non-diabetic non-ischemic limbs supports the ability of this imaging technology to document the effect of diabetes alone to induce RAGE expression in the peripheral arteries.

As occlusive lower extremity lesions progress, flow reserve is lost and there is further progression to resting hypoxia. In the non-diabetic limb, in response to tissue hypoxia, VEGF is released locally via the HIF-1a pathway and stimulates angiogenesis through multiple mechanisms that include increased proliferation and decreased apoptosis of endothelial cells and chemoattraction of monocytes into the ischemic tissue [8,9]. In diabetes, this normally adaptive response to hypoxia is blunted. RAGE has been shown to have a major mechanistic role in this maladaptive response by reducing VEGF mRNA and by inducing a defect in signal transduction of VEGF in monocytes which reduces their response to chemotactic protein-1, resulting in fewer monocytes in the tissue to stimulate angiogenesis [11,12,17]. While the murine femoral artery ligation hind limb ischemia model lacks the component of occlusive proximal arterial disease found in humans, it is currently a standard small-animal model used to study the molecular biology of limb ischemia [18]. The results of the experiments reported in this manuscript concur with the molecular biology reported for RAGE and angiogenesis pathways.

Clinical imaging tests for lower extremity ischemia focus on detection of blood flow reduction and/or presence and location of occlusive arterial lesions [19]. There is potential for a molecular imaging approach targeting the biology of peripheral artery disease to demonstrate the extent of small-vessel disease. Two important targets in this disease process are RAGE expression and its effect on the angiogenic responses to tissue hypoxia. A pathology study reported by Rigghaler et al. in 1995 documented prominent enhancement of endothelial RAGE expression in small- and medium-sized arteries in patients with occlusive peripheral vascular disease both with and without diabetes [20]. While large vessels such as the aorta can be visualized at necropsy, smaller arteries require multiple sampling for histology and quantification is subject to sampling error. With this limitation, it is more difficult to assess the total extent of receptor expression. An *in vivo* molecular imaging approach which can visualize an entire limb has the potential to provide generalized vascular expression of RAGE in live subjects both as a research tool and as a non-invasive marker for the effect of therapies directed at suppressing RAGE to improve the symptoms and prognosis for PAD.

Experimental studies suppressing RAGE ligands have shown improvement in angiogenic response to limb ischemia [13,14]. Drugs that inhibit ligands that bind RAGE have been tried in humans, but each has limitations regarding efficacy or untoward side effects [21]. Aminoguanidine, a small hydralazine-like compound, blocks AGE formation but did not advance in clinical trials due to adverse side effects in diabetics attributed to sequestration of pyridoxal and vitamin B6 deficiency [21]. Another approach is to block AGEs with soluble RAGE to prevent binding to RAGE and suppress initiation of the signaling pathways [22]. Soluble RAGE made from the extracellular two thirds of the receptor was developed for mouse studies, but limited quantities are currently available. A humanized rat monoclonal anti-RAGE antibody that antagonizes RAGE interactions with multiple ligands was shown to be a potent protector against the toxic effects of sepsis [23]. Because of the multiple ligands binding RAGE and its importance in a spectrum of diseases, there is a clear impetus to developing better RAGE-suppressing drugs. A non-invasive tool that can quantify RAGE expression *in vivo* would be useful in monitoring the effects of such drug on the molecular signals.

## Conclusions

The results of these experiments document the feasibility of imaging and quantifying RAGE expression in the hind limbs of a relevant mouse model of limb ischemia and show the effect of diabetes to increase the expression and thereby reduce angiogenesis. Future directions include combined imaging protocols targeting both RAGE and either ανβ_3_ integrin and/or VEGF to more completely map the biology of limb ischemia in the live animal, to follow the mice longer after occlusion, and to monitor the functional consequences of flow reduction using ultrasound techniques.

## Abbreviations

AGEs: Advanced glycation endproducts; DTPA: Diethylene triamine pentaacetic acid; FOV: Field of view; HIF-1a: Hypoxia-inducible factor-1 alpha; HMGB1: High-mobility group box 1; ID: Injected dose; PAD: Peripheral artery disease; RAGE: Receptor for advanced glycation endproducts; SPECT/CT: Single-photon emission computed tomography/computed tomography; STZ: Streptozotocin; VEGF: Vascular endothelial growth factor.

## Competing interests

The authors declare that they have no competing interests.

## Authors’ contributions

YT helped design the experiments, prepared the tracers, and performed the experiments. MK helped in the acquisition of data. LJ helped design the experiments, analyze and interpret data, and prepare the manuscript. GZ performed the tissue staining. AMS helped in the design of the experiments and as consultant. CL helped with the animal experiments and with the anesthesia, injections, imaging, necropsy, and tissue preparation. All authors read and approved the final manuscript.
